# Infection control strategy and primary care assistance in Campania region during the national lockdown due to COVID-19 outbreak: the experience of two tertiary emergency centers

**DOI:** 10.1186/s13052-021-00963-3

**Published:** 2021-01-29

**Authors:** Angela Mauro, Nicola Improda, Letizia Zenzeri, Francesco Valitutti, Erica Vecchione, Sara Esposito, Vincenzo Tipo

**Affiliations:** 1 Emergency Pediatric Department, Pediatric Emergency Unit, AORN Santobono-Pausilipon Children’s Hospital, Via Mario Fiore 6, 80131 Naples, Italy; 2 EBRIS (European Biomedical Research Institute of Salerno), Salerno, Italy; 3 Pediatric Section, Department of Translational Medical Sciences, Federico II University of Naples, Naples, Italy; 4 Pediatric Unit, AOU San Giovanni di Dio e Ruggi D’Aragona, Salerno, Italy

**Keywords:** Triage, Emergency care, COVID-19, Infection control strategy

## Abstract

**Background:**

COVID-19 pandemic has markedly affected emergency care, due to sudden limitation of health care capacity by general practitioners (GP) and urgent need for infection control strategies. We evaluated the activity of the Emergency Department (ED) during the national lockdown (March 8–April 30), as well as the outcomes of our infection control strategy.

**Results:**

Despite a reduction in access by one fifth, a proportion of febrile patients comparable to 2019 was seen (829/2492, 33.3% vs 4580/13.342, 34.3%, *p* = 0.3). Diagnostic swab for COVID-19 was performed in 25% of patients, especially in subjects with co-morbidities or multiple access. Six infected cases were identified, all presenting with febrile disease. Only two positive patients fulfilled the criteria for diagnostic swab provided by the Italian Health Authorities, because of close contact with suspected or confirmed cases.

The rate of admission for febrile or respiratory conditions was higher than the same period of 2019 (33.4% vs 25.9%, *p* < 0.0001). None of the 105 health-care professionals working during the study time lapse exhibited anti-SARS-CoV-2 seroconversion. Among the 589 patients with information available, 54.9% declared no medical consultation at all prior to coming to ED, while only 40 (of which 27 with fever) had been examined by their GP before coming to ED. Nevertheless, 35.6% of the cases were already taking medications. None of the 9 patients requiring intensive care reported recent pediatric consultation, despite symptoms duration up to 30 days.

**Conclusion:**

Our results provide evidence that the reduced capacity of primary care facilities during the national lockdown may have caused a high rate of self-medication as well as a delayed provision of care in some patients. Identification of pediatric patients affected with SARS-CoV-2 infection remains a challenge because of the absence of reliable predictive factors. Finally, the use of specific triage centers, with dedicated pathways to diagnose SARS-CoV-2 infection, trace contacts and allow adequate care after swabs, is effective in preventing spreading of the infection.

## Background

The pandemic of novel coronavirus (severe acute respiratory syndrome coronavirus 2, SARS-CoV-2) causing a cluster of respiratory infections (coronavirus disease 2019, COVID-19) has rapidly transformed the healthcare system of most countries, with emergency representing one of the most affected areas. Being at the forefront of preventing and controlling the spread of SARS-CoV-2 infection, emergency clinicians had to learn quickly as much as possible about SARS-CoV-2, and to keep constantly updated about this new disease. This was made even more challenging by the fact that pediatric patients with confirmed SARS-CoV-2 infection seem to present with mild, nonspecific symptoms [1–4], with epidemiological risk factors (especially the familial context) being of paramount importance to identify affected children [1]. Another important factor influencing emergency care has been the limited possibility of primary care facilities to perform physical evaluation in subjects with febrile or respiratory conditions, due to the lack of appropriate protection equipment and dedicated spaces [5].

**Fig. 1:**
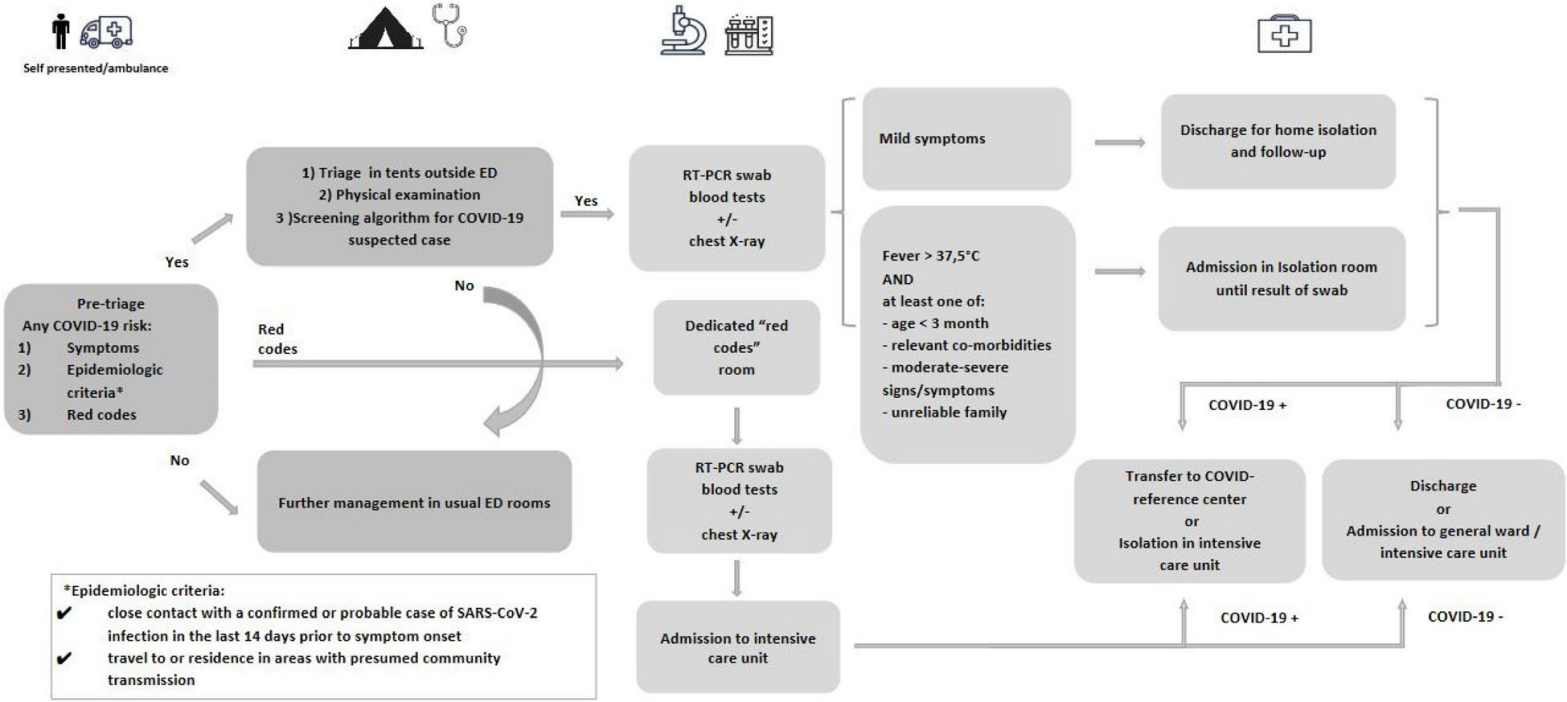
Algorithm for infection control strategy

According to international guidelines [6], our emergency units established specific strategies to detect and isolate children infected with SARS-CoV-2, thus limiting intra- and extra-hospital spreading of the infection. Our infection control strategy included a pre-triage before entering the Emergency Department (ED) hall, a triage for patients with symptoms suggestive of infection in tents located outside the ED and dedicated intra-hospital paths for patients with suspected or confirmed infection (Fig. 1).

We designed this multicenter retrospective study aiming to assess the effects of COVID-19 outbreak on the activity of the ED during the first national lockdown. Additional aim was to evaluate the effectiveness of our integrated infection control strategy in preventing nosocomial infections.

## Methods

### Infection control strategy

AORN Santobono-Pausillipon (Naples) and San Giovanni di Dio e Ruggi D’Aragona University Hospital (Salerno) represent tertiary emergency centers designated as Regional Spoke for screening of children with SARS-CoV-2 infection in Campania region.

The two hospitals have adopted an infection control strategy (Fig. 1) consisting in pre-triage of all cases before entering the ED hall, followed by a triage of all subjects with symptoms suggestive of infection in tents located outside the ED outpatient hall, where the patients also received medical evaluation. Red codes were considered potentially infected and thus were evaluated in a dedicated “red codes” room by designated health-care personnel.

If the screening algorithm for COVID-19 suggested a suspected case, relevant diagnostic procedures (blood tests, diagnostic swab, chest X-ray) were performed into the triage tent or in a dedicated “procedure room” located at the beginning of the intra-hospital COVID path. Diagnostic procedures for red codes were performed directly into the “red codes” room, if possible.

Healthcare personnel were protected by using FFP2/N95 or FFP3 face mask, eye protection goggles, isolation gown and shoes and double gloves. All patients or caretakers were provided with a surgical mask for intra- and extra-hospital transfer.

After the triage, patients without suspicion of SARS-CoV-2 infection were referred to general ED rooms for further management.

Subjects with suspected infection underwent a diagnostic swab. Among them, those requiring hospitalization (fever > 37.5 °C and at least one of: age < 3 months, relevant comorbidities, moderate-severe signs/symptoms, unreliable family) were transferred through a protected path into an isolation room within our COVID-unit/area until the results of the swab came back, while the rest (mild symptoms) was discharged home for isolation with monitoring by the local health services and, if a positive result of the swab returned, was called back for admission to COVID reference center. Given the wide local spreading of SARS-CoV-2 infection at the beginning of March 2020 in Campania, execution of the diagnostic swab was extended also to subjects who did not fulfill the criteria provided by the Italian Health Authorities [7], namely: a) fever and/or respiratory symptoms and close contact with a confirmed or probable case of SARS-CoV-2 infection, or having stayed in areas with presumed community transmission; b) severe acute respiratory illness (SARI) with no clear cause and necessitating hospitalization, or rather subjects coming as red code or, on an individual basis, with unexplained or prolonged fever and/or acute respiratory symptoms (at least one of cough or tachypnea) and/or febrile gastroenteritis.

Patients with confirmed infection were eventually transferred from our COVID-unit to the local Infectious Diseases Services.

Critically ill patients were transferred to the intensive care unit (ICU) of AORN Santobono-Pausilipon, which was identified by the regional health authorities as reference centre for suspected or confirmed COVID-19 cases needing intensive care.

### Study population and data recording

**Fig. 2:**
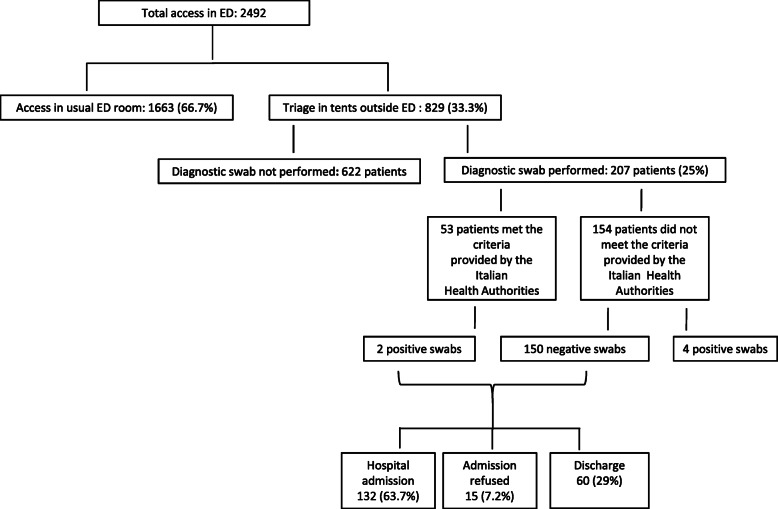
Overview of patients coming to the ED during the national lockdown (from the 9th of March to the 30th of April 2020)

We retrospectively reviewed files of children and adolescents aged up to 14 years who accessed the EDs of the two hospitals from the 8th of March to the 30th of April 2020. We then extrapolated data from patients evaluated in the triage tents or presenting as red code (Fig. 2). All patients were triaged by specialized nurses and were assigned in 4 class of severity: 1) red (resuscitation); 2) yellow (urgent care); 3) green (less urgent care); 4) white (non-urgent care) [8].

Both epidemiological (close contact with family members or subjects with suspected or confirmed infection, and residence or traveling into or coming from a high prevalence area of SARS-CoV-2 infection) and clinical (vital signs and symptoms) data were recorded.

Involvement of emergency medical transports services (i.e. ambulances), duration of symptoms, need for chest x-ray, previous medical consultation, ongoing treatments, modalities of treatment prescription, and need for intensive care were recorded, aiming to evaluate primary health care assistance of children with fever or respiratory symptoms during the pandemic.

All the healthcare personnel involved in the COVID pathway (doctors, nurses and health care assistants) underwent an infection control surveillance consisting in systematic check before entering the hospital for body temperature and suggestive symptoms, as well as regular check for SARS-CoV-2-specific IgM and IgG every 15 to 20 days. In order to assess the efficacy of the infection control strategy, the results of all serological tests performed during the observation period were collected and analyzed.

Data were retrieved from an audit performed within the two hospitals. Databases were anonymized.

### Diagnostic procedures

Confirmation of SARS-CoV-2 infection was obtained via a real-time polymerase chain reaction (RT-PCR) for SARS-CoV-2 nucleic acids on nasal + oropharyngeal swab. RT-PCR was performed at the local CDC reference laboratories (Cotugno Hospital, Federico II University Hospital or Salerno University Hospital).

SARS-CoV-2-specific IgM and IgG were detected by Magnetic Chemiluminescence Enzyme Immunoassay using commercial kits.

### Statistical analysis

Statistical analysis was performed using IBM SPSS Statistics (IBM Corp., Released 2010; IBM SPSS Statistics for Windows, NY, USA). Data are presented as absolute numbers, percentage, means and standard deviations. Student’s T or Chi squared tests were used to compare subgroups data as appropriate. A *p* value < 0.05 was considered significant for all the tests performed.

## Results

Total access in ED among the two hospitals during the study time lapse were 2492, about one fifth of those recorded in the same period of 2019 (13342), as shown in Fig. 2.

**Fig. 3:**
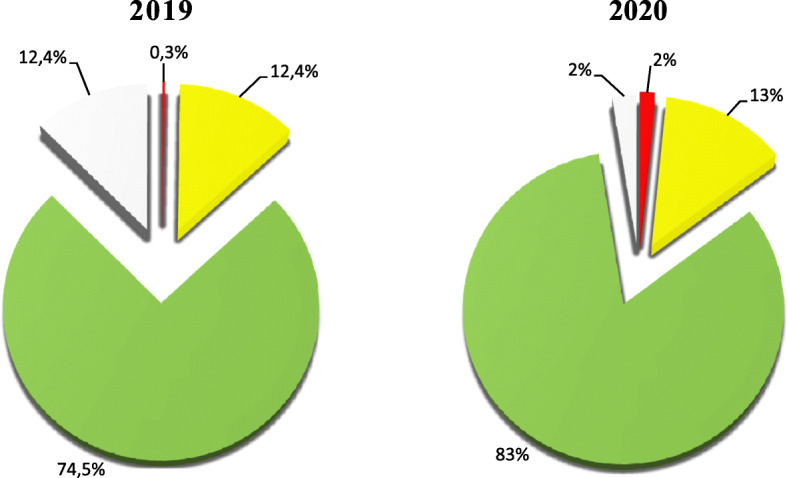
Comparison of distribution of emergency codes reflecting 4 class of severity (red = resuscitation, yellow = urgent care, green = less urgent care, white = non-urgent care) between the same time lapse in 2019 and 2020

Overall, 829 (33.3%) subjects (451 males, age range 4 days-13.9 years) were triaged in the tents because of symptoms suggestive of infection (Fig. 2), with 527 (63.6%) patients younger than 3 years of age. General features of our study cohort are summarized in Table 1. Compared to the same period of last year the proportion of access for fever and/or respiratory symptoms was similar (829/2492, 33.3% in 2020 vs 4580/13342, 34.3% in 2019, *p* = 0.3). As far as it concerns severity 13 red, 109 yellow, 686 green and 21 white codes were seen, with 61 patients (7.4%) coming by emergency medical transports services. Compared to the same period of 2019, as expected the rate of white codes was significantly reduced (2.5% in 2020 vs 12.4% in 2019, *p* < 0.0001), while interestingly the rate of red codes was significantly higher (2% in 2020 vs 0.3% in 2019, *p* < 0.0001) (Fig. 3).

**Table 1:** General characteristics of the study population

Features	Overall cohort	Subjects with co-morbidities
Number of patients (males)	829 (451)	135 (72)
Age (mean ± SD) in years	3.4 ± 3.6	4.5 ± 3.9
Use of emergency medical transports	61	20
Symptoms		
● Fever	608	87
● Cough	445	78
● Dyspnoea	176	45
● Vomiting	100	14
● Diarrhea	77	10
Mean duration of symptoms (days)	3.4 ± 5	2.6 ± 3.6
Epidemiologic features:		
● Travel in high prevalence areas	10	0
● Close contact with suspected (including symptomatic family members) or confirmed cases	30	8
Diagnostic swab	207	39
Criteria for diagnostic swab	53	8
Hospital admission	277	58
Refused admission	32	9

Pharyngeal and nasal swab for exclusion of SARS-CoV-2 infection was performed in 207 patients (25%), of which 53 met the criteria provided by the Italian Health Authorities and, among these, 40 (75.5%) had epidemiological criteria of suspicion (40 (19.3%) had epidemiological criteria of suspicion) (Fig. 2). Two patients among those who met the criteria provided by the Italian Health Authorities (*n* = 53) had a positive diagnostic swab (Fig. 2) and both of these subjects had a close contact with a suspected case of COVID-19. All the patients with confirmed infection had fever with 5/6 (83.3%) cough, one required admission to ICU for concomitant sepsis. Furthermore, the average age of patients with confirmed infection was lower than the total of patients with fever or respiratory symptoms. One hundred thirty-five patients (16.3%) had at least one co-morbidity (Table 1), six of them presented as red codes. The percentage of subjects undergoing a diagnostic swab did not differ between subjects with co-morbidities and the rest of the subjects (39/135, 28.8% vs 170/694, 24.4%, *p* = 0.2).

Information regarding primary health care assistance (Table 2) was available only for 598/829 patients. Only 40 patients (27 with fever) claimed to having been physically seen by their general practitioner (GP), while 94 patients had phone call consultations, and 455 (54.9%) declared no consultation at all prior to coming to ED. Medical treatment (at least one of antibiotics, oral steroids and aerosol) had been already started in 295 cases (35.6%), of which 154 (52.2%) had not been seen by GP and 54 (18%) received a prescription over the phone. Mean duration of symptoms was significantly higher in treated (*n* = 295), compared with untreated (*n* = 534) subjects (5.3 ± 5.6 vs 2.48 ± 4.1 days, *p* < 0.0001).

**Table 2:** Medical assistance before coming to Emergency Department

	Number	%
Patients with data available	598	72.1
Previous examination by GP	40	4.8
Previous phone call consultation with GP	94	11.3
Already taking medications	295	35.6
Medication prescribed by phone	54	18.0
On treatment despite no previous consultations	154	52.2

Compared with the same period of last year, we observed a higher percentage of hospital admission (277/829, 33.4% in 2020 vs 1241/4782, 25.9% in 2019, p < 0.0001), but similar rate of admission refusals (32/829, 3.8% in 2020 vs 155/4782, 3.2% in 2019, *p* = 0.3). The mean time between triage in tent and hospital admission was 353.5+/− 201.7 min (range 9–464). Nine patients were eventually admitted to ICU, but none of them reported recent pediatric consultation, despite mean duration of symptoms 5.9 ± 9.2 days (range 1–30 days).

Multiple access were recorded in 47 patients (Table 1), of which only 3 had been examined by their GP and 14 had a phone call consultation. Patients with multiple accesses exhibited a higher rate of swab for SARS-CoV-2 and hospital admission compared to single access (27/47, 57.4% vs 181/726, 24.9%, *p* < 0.0001 and 33/47, 70.0% vs 246/726, 33.9%, *p* < 0.0001 respectively).

A total number of 105 healthcare professionals (42 doctors, 49 nurses, 14 health-care assistant) was involved into the COVID path during the study time lapse. Each subject was tested for SARS-CoV-2 specific antibodies at least twice and none of them exhibited anti-SARS-CoV-2 seroconversion.

## Discussion

To our knowledge, this is the first study from the South of Italy evaluating the consequences of the reduced health care capacity of pediatric primary care during the national lockdown on the activity of pediatric ED.

Since December 2019, SARS CoV-2 infection has spread rapidly worldwide and on 11 March 2020 the World Health Organization (WHO) classified COVID-19 as a pandemic. Italy is one of the European countries most affected by COVID-19 infection, with a dizzying increase of affected cases since February 2020, inducing the government to establish a national lockdown from the 9th of March and until the 3rd of May, limiting the movement of the population except for necessity [9].

Due to sudden onset of the pandemic, during the lockdown primary health care facilities and GPs had to face a lack of appropriate individual protection equipment and dedicated spaces or protocols. Thus, they started using telephone consultations or digital tools (ie WhatsApp) to triage patients, in order to assess whether it was necessary and safe to visit the patient or if the patient had to be referred to ED or other local health services for screening of SARS-CoV-2 infection or further management [10]. As a result, in most cases GPs were unable to examine children with flu-like symptoms until SARS-CoV-2 infection was ruled out and provided medical advice throughout phone calls or digital tools.

However, our data indicate that many patients continued to seek direct medical consultation in the ED, instead of getting in touch with their GP or waiting for a swab from the local health services. Indeed, despite a drastic reduction of the total access compared to the previous year, a similar proportion of subjects with febrile or respiratory conditions came to ED, in particular subjects with coexisting conditions, which are known to make the patient more vulnerable to severe forms of the infection [11]. Moreover, limited access to physical examination and/or fear of SARS-CoV-2 have most likely contributed to the significant rate of self-medication and of multiple access to ED, especially in patients with long duration of symptoms. Finally, given that a higher rate of red codes was observed and that none of the patients needing intensive care had been previously seen by GP despite significant duration of symptoms, in agreement with other studies [12] we could speculate that fear of SARS-CoV-2 infection might have led to a delayed provision of care in some cases.

Up until the 28th of April, in Italy about 20,000 healthcare workers have tested positive for SARS-CoV-2, accounting approximately for 12% of the national cases [13]. Therefore, a great effort has been made to identify adequate infection control strategies, including accurate protocols for patient management and periodical screening program for healthcare workers [14, 15].

Several studies from different countries [5, 16–18] indicate that triage and work-up in specific areas outside the classical ED, as well as the use of complete protection equipment please, remove s play a key role in reducing intra-hospital spreading of SARS-CoV-2 infection.

Similarly, despite limitation due to the use of only serological tests for healthcare personnel, the results of our study indicate that a strategy consisting in pre-triage and triage carried out in designated areas outside the hospital, the systematic use of DPIs and dedicated pathways for patients with suspected infection is effective in limiting the intra-hospital spreading of SARS-CoV-2 infection.

Interestingly, we observed a higher rate of hospital admission compared to the same period of previous year, regardless of SARS-CoV-2 positivity, suggesting that a well-structured infection control strategy might also have helped in avoiding refusal of hospitalization due to the fear of getting nosocomial SARS-CoV-2 infection. Whether this has also led to an over-exposure to diagnostic or therapeutic procedures needs to be determined in further studies. However, the large time intervals between pre-triage and eventual hospitalization indicate a significant prolongation of stay in isolation rooms identified over the COVID-pathway up to 7 h, due to the number of tests included into our emergency COVID-protocol (swab and serology for screening of SARS-CoV-2, blood tests and/or chest x-ray).

Finally, the results of our study confirm previous data [19–23] indicating that SARS–CoV-2 infection in children has been infrequent (3% rate of positive swabs) during lockdown. When present, mild non-specific symptoms were its constant, with fever being the most frequent sign.

A detailed description of the 6 positive cases is beyond the scope of the study. However, only two of the 6 patients with confirmed infection met the criteria provided by the Italian Health Authorities [7], as they had a close contact with a suspected case of COVID-19,  family members with suggestive symptoms, who had not performed a diagnostic swab. These considerations confirm that besides the classical epidemiological risk factors, a low threshold of suspicion should be kept in regions with a high prevalence of SARS-CoV-2 infection.

## Conclusions

Our study depicts the activity of the only two tertiary EDs in Campania region during  the first national lockdown due to SARS-CoV-2 pandemic. Our results indicate that during the national lockdown ED was considered as a safe and quick option to get a direct medical consultation. We aknowledge that a weakness of our study is mainly inherent to its retrospective design. Nevertheless, we provide clear evidence that the reduced capacity of primary care physicians may have caused a high rate of self-medication as well as a delayed provision of care in some patients. Identification of pediatric patients affected with SARS-CoV-2 infection remains a challenge because of the absence of reliable predictive factors. Finally, the use of specific triage centers, either close or far from the EDs, with dedicated pathways to diagnose SARS-CoV-2 infection, trace contacts and allow adequate care after swabs, is effective in preventing spreading of the infection and could thus guarantee high care standards.

## Data Availability

The datasets used and/or analysed during the current study are available from the corresponding author on reasonable request.

## Abbreviations

GP: General practitioner
ED: Emergency department
SARS-CoV-2: Severe acute respiratory syndrome coronavirus 2
COVID-19: Coronavirus disease 2019
RT-PCR: Real-time polymerase chain reaction
ICU: Intensive care unit
